# Tau Protein and Its Role in Blood–Brain Barrier Dysfunction

**DOI:** 10.3389/fnmol.2020.570045

**Published:** 2020-09-30

**Authors:** Alena Michalicova, Petra Majerova, Andrej Kovac

**Affiliations:** ^1^Institute of Neuroimmunology, Slovak Academy of Sciences, Bratislava, Slovakia; ^2^Department of Pharmacology and Toxicology, The University of Veterinary Medicine and Pharmacy, Kosice, Slovakia

**Keywords:** neurovascular unit, blood-brain barrier, tauopathy, tau protein, neuroinflammation

## Abstract

The blood–brain barrier (BBB) plays a crucial role in maintaining the specialized microenvironment of the central nervous system (CNS). In aging, the stability of the BBB declines and the permeability increases. The list of CNS pathologies involving BBB dysfunction is growing. The opening of the BBB and subsequent infiltration of serum components to the brain can lead to a host of processes resulting in progressive synaptic, neuronal dysfunction, and detrimental neuroinflammatory changes. Such processes have been implicated in different diseases, including vascular dementia, stroke, Alzheimer’s disease (AD), Parkinson’s disease, multiple sclerosis, amyotrophic lateral sclerosis, hypoxia, ischemia, and diabetes mellitus. The BBB damage is also observed in tauopathies that lack amyloid-β overproduction, suggesting a role for tau in BBB damage. Tauopathies represent a heterogeneous group of around 20 different neurodegenerative diseases characterized by abnormal deposition of the MAPT in cells of the nervous system. Neuropathology of tauopathies is defined as intracellular accumulation of neurofibrillary tangles (NFTs) consisting of aggregated hyper- and abnormal phosphorylation of tau protein and neuroinflammation. Disruption of the BBB found in tauopathies is driven by chronic neuroinflammation. Production of pro-inflammatory signaling molecules such as cytokines, chemokines, and adhesion molecules by glial cells, neurons, and endothelial cells determine the integrity of the BBB and migration of immune cells into the brain. The inflammatory processes promote structural changes in capillaries such as fragmentation, thickening, atrophy of pericytes, accumulation of laminin in the basement membrane, and increased permeability of blood vessels to plasma proteins. Here, we summarize the knowledge about the role of tau protein in BBB structural and functional changes.

## Introduction

Tau proteins are the most frequent microtubule-associated proteins in the brain and are characterized as intrinsically disordered proteins. They are abundant in the neurons of the central nervous system (CNS) and have roles primarily in maintaining the stability of microtubules in axons. They are also less expressed in brain-resident immune cells: astrocytes and oligodendrocytes (Williams, 2006). Tau proteins are important for cell signaling, synaptic plasticity, and regulation of genomic stability (Guo et al., 2017). The adult human brain expresses at least six isoforms of tau protein, which are derived from a mictorubule-associated protein tau (MAPT) tau gene as a result of alternative splicing of its messenger RNA (Goedert et al., 1989; Hanes et al., 2009; Zhang et al., 2009). The tau isoforms range in size from 352 to 441 amino acid residues and differed on the presence of 0, 1, or 2 sequence inserts in the amino-terminus of the protein and inclusion or exclusion of the second of four microtubule-binding potential repeat domains coded by exon 10 (Hanes et al., 2009; Grinberg et al., 2013). In humans, tau is subject to many post-translational modifications, including hyperphosphorylation, truncation, nitration, glycation, glycosylation, ubiquitination, polyaminations, and self-aggregation into insoluble paired helical filaments (Mena et al., 1996; Mohandas et al., 2009; Novak, 2012; Beharry et al., 2014).

Tauopathies represent a heterogeneous group of around 20 neurodegenerative diseases characterized by abnormal deposition of the MAPT in neurons and glial cells (Zilka et al., 2009; Ferrer et al., 2014). Histopathologically, the tauopathies are characterized by the presence of intracellular insoluble inclusions of abnormally modified protein tau into neurofibrillary or gliofibrillary tangles. Tangles are formed by hyperphosphorylated tau protein, causing the tau to dissociate from microtubules and form insoluble inclusions. Tauopathies are classified by the predominance of tau isoforms found in cytoplasmic inclusions of tau protein: those with inclusions predominantly composed of tau with 3-repeat (3R-tauopathies), those with predominantly 4-repeat (4R-tauopathies), or an equal ratio of 3R:4R tau. The most common tauopathies are Alzheimer’s disease, frontotemporal dementia with parkinsonism liked to chromosome 17 (FTDP-17), progressive supranuclear palsy (PSP), corticobasal degeneration (CBD), and Pick’s disease (PiD). There are also rarer tauopathies including argyrophilic grain disease (AGD), postencephalitic parkinsonism (PEP), parkinsonism dementia complex of Guam (PDCG), tangle-dominant dementia, and a new category of tauopathy known as the globular glial tauopathies (GGTs). According to their tau pathology, we can divide tauopathies into three main groups (Table 1).

**Table 1:** The first subgroup of tauopathies is represented by 3R/4R tauopathies, such as Alzheimer’s disease (AD), frontotemporal dementia, and parkinsonism linked to chromosome 17, parkinsonism dementia complex of Guam (Lytico-bodig disease), chronic traumatic encephalopathy, postencephalitic parkinsonism, atypical parkinsonism of Guadeloupe, primary age-related tauopathy, or diffuse neurofilament tangles with calcification.

Disease	Predominant isoform	Blood–brain barrier changes	Amyloid-β deposition	References
Alzheimer’s disease	3R + 4R	Changes in the regulation of cerebral blood flow Increase in endothelial pinocytosis Decrease in mitochondrial content Accumulation of collagen Loss of tight junctions leading to the BBB breakdown and subsequent infiltration of blood-borne molecules Failure of influx and efflux transport systems Altered levels of agrin Upregulation of aquaporin AQP4 expression Increased numbers of fragmented vessels with fewer intact branches Atrophic string vessels Irregularities in the capillary surface Thickening, vacuolization, and local disruption of the capillary basement membrane Accumulation of laminin in basement membranes Atrophy and increased numbers of pericytes Swelling of astrocytic endfeets Loss of the perivascular plexus Vascular smooth muscle actin reduction Loss of tight junctions proteins	Present	Higuchi et al. (1987), Scheibel et al. (1987), Miyakawa et al. (1988), Stewart et al. (1992), Buee et al. (1997), Zlokovic (2005), Abbott et al. (2006), Desai et al. (2007), Pérez et al. (2007), Liu et al. (2008), Zlokovic (2011), Østergaard et al. (2013), Cai et al. (2018), and Yamazaki et al. (2019)
Frontotemporal dementia and parkinsonism linked to chromosome 17	3R, 4R/3R + 4R	Microbleeds predominantly within the areas affected by neuronal pathology	Absent	De Reuck et al. (2012) and De Reuck et al. (2013)
Progressive supranuclear palsy	4R	Decreased P-gp function in basal ganglia and frontal region	Absent	Bartels et al. (2008)
Corticobasal degeneration	4R	Few cases of vascular CBS with multiple infarcts in frontal lobe, motor cortex, periventricular white matter, thalamus, basal ganglia and corticospinal tract degeneration	Absent	Koga et al. (2019)
Chronic traumatic encephalopathy	3R + 4R	BBB disruption in regions of intense perivascular tau deposition Loss of claudin-5 and enhanced extravasation of endogenous blood components such as fibrinogen and IgG	Present in approximately half of the cases	McKee et al. (2009) and Farrell et al. (2019)
Parkinson–dementia complex of Guam (Lytico-bodig disease)	3R + 4R	Decreased vascular density Thin and atrophic microvessels with severe fragmentation resulting in a reduced number of microvascular branches String vessels localized mainly in the areas affected by NFTs pathology Upregulation of adhesion molecules Disruption of tight junctions Increase of collagen-type IV content per vessel	Present in some cases	Buee et al. (1994), Buee et al. (1997), and Majerova et al. (2018)
Pick disease	3R	Ramified astrocytes Thinning of microvessels Increased tortuosity Twisted vessels Fragmentation of microvasculature in atrophy affected brain regions	Absent	Buee et al. (1994), Komori (1999), and Buee et al. (1997)
Argyrophilic grain disease	4R	-	Absent	-
Globular glial tauopathies	4R	Mild BBB disruption in mice after the targeted expression of human wild-type tau in murine astrocyte Astrocytic modulation of synaptic function by the secretion and uptake of neurotransmitters such as glutamate	Absent	Zlokovic (2008), Bartels et al. (2009), and Haydon (2001)
Aging-related tau astrogliopathy	4R	Possible functional changes of the BBB	Absent	Kovacs et al. (2016)
Primary age-related tauopathy	3R + 4R	-	Absent	-
Postencephalitic parkinsonism	3R + 4R	-	Very rare	-
Atypical parkinsonism of Guadeloupe	3R + 4R	-	Absent	-
Diffuse neurofibrillary tangles with calcification	3R + 4R	-	Absent	-

*Three-repeat tauopathies include Pick’s disease and some forms of frontotemporal dementia and parkinsonism linked to chromosome 17 and to the four-repeat tauopathies belong progressive supranuclear palsy, corticobasal degeneration, argyrophilic grain disease, globular glial tauopathies, or aging-related tau astrogliopathy*.

1. 3R tauopathies
2. 4R tauopathies
3. 3R/4R tauopathies

However, tau neuropathology is rarely isolated, and it is associated with the deposition of at least one other amyloidogenic protein, such as α-synuclein or huntingtin in most tauopathies. Based on this, we can assume, that tau may have important pathological roles in these disorders with multiple pathologies (Jensen et al., 1999; Hashiguchi et al., 2000).

AD, representing around 70% of all dementia cases, is the best-described tauopathy (Avila et al., 2004). Brain pathology in AD is characterized by the presence of extracellular amyloid-β plaques and intracellular (NFTs; Glenner and Wong, 1984; Grundke-Iqbal et al., 1986). For more than 20 years, the research has been focused on “amyloid-β cascade hypothesis” (Armstrong, 2013), according to which, the amyloid-β is the central pathological feature of AD. This theory assumed that the elimination of amyloid-β could be therapeutic in AD patients. This theory was mainly supported by the discovery of a familial form of AD, which is connected with an APP gene mutation (Korczyn, 2008). The majority of Alzheimer’s patients do not have a mutation causing an increase in APP; therefore, other processes, such as the impaired elimination of amyloid-β, can cause the accumulation of amyloid-β (Preston et al., 2003; Bell and Zlokovic, 2009). On the other hand, the “tau hypothesis” is based on a strong correlation between the clinical symptoms of AD and neurofibrillary pathology. Under pathological conditions, highly soluble tau protein undergoes different post-translational with the latter formation of insoluble paired helical filaments (Mena et al., 1996; Mohandas et al., 2009). These processes lead to the neuronal death and subsequent release of pathologically modified tau proteins into the extracellular environment, activating microglia and inducing the spread of tau pathology by a prion-like mechanism (Maccioni et al., 2010; Kovac et al., 2011). These facts clearly demonstrate that unlike amyloid-β senile plaques, tau pathology correlates with the progress of AD, and therefore, it is considered by many to be the main cause of neurodegeneration (Kovacech et al., 2009).

## Neurovascular Changes in Tauopathies

CNS is considered to be one of the most delicate systems in the human body. This fact explains the necessity to maintain the extracellular environment of the CNS highly regulated (Hawkins and Davis, 2005). Three main barrier layers at the interface between blood and tissue protect the CNS: (i) the choroid plexus epithelium located between the blood and the ventricular cerebrospinal fluid; (ii) the arachnoid epithelium situated between the blood and the subarachnoid cerebrospinal fluid; and (iii) the vascular blood-brain barrier (BBB), which mediates the communication between the periphery and the CNS (Abbott, 2005).

BBB strictly controls the exchange of cells and molecules between blood and CNS. Previously, the BBB has been characterized as a layer of endothelial cells forming the vessel/capillary wall. Recently, the BBB is a component of the neurovascular unit (NVU: Abbott et al., 2006). The NVU represents a highly dynamic system, where the proper functioning of the brain depends on the functional interactions of the endothelial cells, neurons, pericytes, mast cells, and glial cells. Moreover, the NVU can be expanded to include circulating immune cells and peripheral tissue cells, which are connected to it by humoral secretions and thus can influence the physical, biochemical, and immune processes of the CNS barriers (Kousik et al., 2012; Wong et al., 2013).

**Figure 1:**
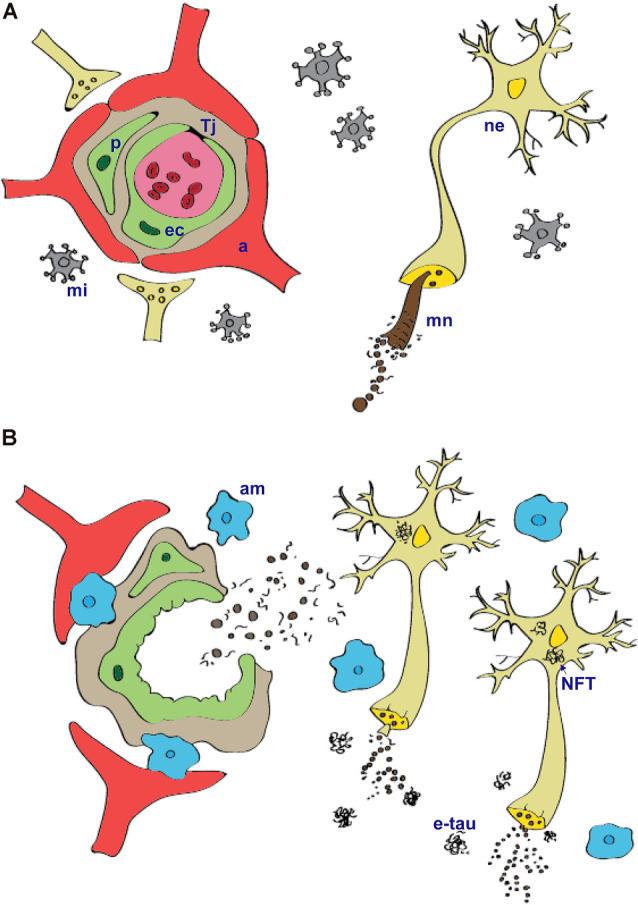
Tau protein physiology **(A)** and pathology **(B)** within the neurovascular unit. Microtubule-associated protein tau is important for assembly and stabilization of microtubular network (mn) in the neurons (ne). In tauopathies, abnormally modified tau protein that is hyperphosporylated and truncated form insoluble intracellular aggregates—neurofibrillary tangles. Microtubular network in axons disintegrate, and this leads to impairment in communication between neurons. Injured neurons and extracellular tau aggregates (e-tau) signal to the microglia (mi) that become activated. Activated microglia (am) produce pro-inflammatory cytokines and chemokines that can cause structural and functional changes of the blood–brain barrier. Prolonged local neuroinflammation stimulate transmigration of peripheral blood monocyte-derived macrophages and release of plasma components into extravascular space. a, astrocytes; Tj, tight junctions; ne, neurons; ec, endothelial cells; p, pericytes; mi, microglia; mn, microtubular network; am, activated microglia; NFT, neurofibrillary tangles; etau, extracellular tau aggregates.

The BBB is formed by endothelial cells firmly attached by tight junctions that restrict the paracellular pathway (Stamatovic et al., 2008). Intercellular junctions play crucial roles in tissue integrity and also in vascular permeability (Wallez and Huber, 2008; Zlokovic, 2008; Tietz and Engelhardt, 2015). Endothelial cells are closely surrounded by pericytes and enclosed by the basement membrane. Pericytes occupy the perivascular space between the capillary wall and astrocytic endfeet, except in the large vessels where smooth muscle cells replace them. Astrocytic endfeet surround the endothelial cells of the BBB and provide biochemical support (Figure 1A). The BBB phenotype has developed mainly under the influence of astrocytes and pericytes and is generally characterized by more complex tight junctions than in other endothelial cells and a number of specific transport and enzyme systems that are responsible for the transport of molecules across the endothelial cells (Abbott, 2002; Bell et al., 2010). The physical breakdown of the BBB is caused by disruption of cell-to-cell junctions between endothelial cells, rising bulk-flow fluid transcytosis, and/or enzymatic degradation of the capillary basement membrane (Zlokovic, 2011).

Paracellular and transcellular pathways are the most common ways for the transport of molecules across the BBB. During the paracellular pathway, molecules are transported between the endothelial cells through the junctional complex. The paracellular pathway is described as a passive transport based on the movement of hydrophilic molecules across the barrier, depending on their electrochemical, hydrostatic, and osmotic gradient (Fu and Wright, 2018). The transport is controlled by tight junctions regulated by various signaling cascades. The principal integral membrane tight junction proteins are occludin, claudin-1, claudin-5, and members of the JAMs family. These proteins interact with other tight junctions proteins, such as ZO-1, ZO-2, and cingulin in the cytoplasm. Subsequently, they are connected to actin filaments (Di Liegro and Savettieri, 2005).

Transcellular transport provides the movement of nutrients, ions, or particles across the BBB depending or not depending on energy. During the transcellular transport, molecules are transported across the luminal and abluminal membrane of the capillary endothelium by different mechanisms including receptor-mediated transcytosis, efflux transport systems, endocytosis of positively charged molecules, and carrier-mediated transport (Fu and Wright, 2018).

The list of CNS pathologies involving BBB dysfunction is rapidly expanding. BBB disruption is associated with numerous pathological conditions that affect the CNS such as ischemia, infections, epilepsy, tumors, and neuroinflammatory diseases including tauopathies. AD is the only tauopathy with described mechanisms of how NVU changes contribute to its pathology. Few reports characterize the general morphology of vessels in PiD, PDCG, FTDP-17, traumatic brain injury/chronic traumatic encephalopathy (CTE), and vascular PSP. In case of PSP, GGTs, and aging-related tau astrogliopathy, a few reports describing functional changes of NVU exists. For other tauopathies, there are no reports dealing with vascular or NVU changes (Michalicova et al., 2017).

### The Role of Tau in the Regulation of BBB Integrity in Physiological and Pathological Conditions

The “healthy” BBB is crucial for proper neuronal function and “healthy” neurons are important for maintaining local milieu of NVU (Rhea et al., 2019). Neurons are very closely associated with brain capillaries. It was estimated that almost every neuron has its own capillary (Zlokovic, 2008). Neurons are important for maintaining the tight junctions (Savettieri et al., 2000), metabolism, regulation of blood flow, and permeability (Zvi et al., 1997). Thus, pathological processes in neurons inevitably affect the NVU.

That the neuropathological hallmarks such as intra- and extracellular protein aggregates are associated with chronic neuroinflammation has long been known. Chronic neuroinflammation affects the BBB by increasing vascular permeability, promoting structural changes in brain capillaries such as fragmentation, thickening, atrophy of pericytes, accumulation of laminin in the basement membrane, increasing permeability to small molecules and plasma proteins, enhancing the migration of immune cells, altering transport systems, or influencing the role of BBB as signaling interface (De Vries et al., 2012; Erickson and Banks, 2013b; Persidsky et al., 2016; Figure 1B). The inflammatory mediators play an important role in regulating blood-to-brain cell transmigration, perpetuating inflammation, and thus exacerbating the disease pathology. Structural and functional changes of BBB lead to progressive synaptic and neuronal dysfunction (Di Liegro and Savettieri, 2005; Zlokovic, 2008; Abbott et al., 2010).

Previously, it has been shown that tangle formation correlated with neuroinflammation in AD (Dipatre and Gelman, 1997; Overmyer et al., 1999; Sheffield et al., 2000; Laurent et al., 2018) and in non-AD human tauopathies such as tangle-predominant dementia, Guam parkinsonism dementia, PSP, and CBD (Imamura et al., 2001; Ishizawa and Dickson, 2001). Moreover, neuroinflammation linked to tau deposition has been well documented in mice transgenic for human mutant tau protein (Bellucci et al., 2004; Yoshiyama et al., 2007) or in transgenic rats expressing misfolded truncated tau protein derived from AD (Zilka et al., 2006; Stozicka et al., 2010).

We showed that in contrast to amyloid-β peptides, truncated tau is not directly toxic to brain endothelial cells. The effect of tau is however mediated through the activation of glial cells (Kovac et al., 2009). Moreover, tau-induced activation of glial cells increased expression of endothelial adhesion molecules and increased transport of leukocytes across BBB (Majerova et al., 2019).

Together, these findings suggest that tau protein has an important role in regulation of microenvironment within the NVU during the physiological and pathological conditions.

### Alzheimer’s Disease

AD is a globally widespread chronic disease affecting around 25 million people worldwide. AD is characterized by cerebrovascular and neuronal dysfunctions leading to a progressive decrease in cognitive functions (Bell and Zlokovic, 2009; Dalvi, 2012). On the neuropathological level, AD is defined by the presence of extracellular amyloid plaques composed of amyloid-β peptide aggregates and NFTs formed by hyperphosphorylated, truncated, and aggregated tau protein, neural loss, loss of synapses, neuroinflammation, and oxidative stress (Cai et al., 2018). The neurofibrillary pathology in AD contains insoluble inclusions of 3R and 4R tau isoforms (Siddiqua and Margittai, 2010).

Two basic forms of AD are known. Familial cases are predominantly early-onset (younger than 65 years), but also late-onset cases have been described (Bekris et al., 2010). This type is usually caused by specific mutations in transmembrane proteins—amyloid precursor protein (APP), presenilin 1, or presenilin 2. The latter two proteins are essential components of a protease complex involved in the generation of amyloid-β from APP (Bertram et al., 2010; Iqbal et al., 2014; Wirz et al., 2014). The late-onset (older than 65 years) sporadic form is more complex and representing more than 99% of all cases (Bertram et al., 2010). The presence of several additional risk factors was described, including traumatic brain injuries (Walter and van Echten-Deckert, 2013), diabetes mellitus, hypercholesterolemia, inflammation, environmental factors, such as diet, toxicological exposure, hormonal factors, and various aspects of lifestyle (Lahiri et al., 2007; Stozicka et al., 2010). Moreover, evidence suggests that the BBB dysfunction is one of the most common pathophysiological hallmark of AD involved in vascular risk factors (Tarasoff-Conway et al., 2016; Yamazaki and Kanekiyo, 2017; Miners et al., 2018). In AD, the functional and structural changes of BBB have been investigated for more than 30 years (Erickson et al., 2012). De La Torre reviewed more than 200 studies showing vascular involvement in AD. According to his results, he concluded that cognitive impairment and CNS pathology might be secondary to vascular changes in AD (De La Torre, 2002).

The leakage of substances from plasma into the CNS, changes in efflux and influx transporters leading to accumulation of the toxins in the CNS, and altered expression and secretion of proteins by the NVU cells are the critical pathways of vascular dysfunction connected with AD (Deane and Zlokovic, 2007; Lok et al., 2007; Neuwelt et al., 2008; Bell and Zlokovic, 2009; Bell et al., 2010; Zlokovic, 2011; Erickson and Banks, 2013a). In AD patients, changes in the regulation of cerebral blood flow caused by reduced microvascular density, an increase in endothelial pinocytosis, decrease in mitochondrial content, accumulation of collagen, and loss of tight junctions leading to the BBB breakdown and subsequent infiltration of blood-borne molecules are frequently present (Zlokovic, 2011; Østergaard et al., 2013). Thanks to the short distance between adjacent brain capillaries, a rapid exchange of substances between the CNS and blood circulation can occur. Malfunction of the nutrient and oxygen supply or impaired elimination of toxic metabolic waste can cause dysregulations of neuronal functioning. Often degeneration of brain endothelial wall in AD results in the accumulation of amyloid-β on the outer side of the basement membrane, promoting a local neuroinflammatory vascular response (Zlokovic, 2005). Failure of the amyloid-β transport from brain to the periphery is caused especially by the decreased levels of LRP-1 (low-density lipoprotein receptor-related protein 1) and increased levels of RAGE (receptor for advanced glycation end products; Cai et al., 2018). Activated NVU cells start to release pro-inflammatory cytokines and vasoactive substances. Subsequent processes, such as the decrease of cerebral blood flow and amplification of cells, contribute to cognitive impairment. Zlokovic suggests that such physiological changes of the NVU, compromised brain microcirculation, and vascular neuroinflammatory responses play an important role in the development of AD (Zlokovic, 2005). Other functional changes of brain vasculature in AD include failure of influx (glucose transporters GLUT1 and GLUT3) and efflux transport systems (P-glycoprotein), altered levels of agrin, and upregulation of aquaporin AQP4 expression (Abbott et al., 2006; Desai et al., 2007; Liu et al., 2008). Moreover, in some AD brains, structural changes of the vasculature, such as increased numbers of fragmented vessels with fewer intact branches, atrophic string vessels, different irregularities in the capillary surface, changes of vessel diameter, thickening (Zlokovic, 2005), vacuolization (Buee et al., 1997), and local disruption of the capillary basement membrane have been observed. Increased numbers of pericytes (Stewart et al., 1992), atrophy of pericytes (Miyakawa et al., 1988), swelling of astrocytic endfeet (Higuchi et al., 1987), loss of the perivascular plexus (Scheibel et al., 1987), vascular smooth muscle actin reduction, and accumulation of laminin in basement membranes have also been reported (Pérez et al., 2007). Pericytes work as gatekeepers of the BBB, and in cooperation with other NVU cells, they regulate the transport of nutrients and waste products between the interstitial fluid and peripheral blood (Thomsen et al., 2017). Multiple studies implicate the connection between pericytes, BBB dysfunction, and neurologic diseases (Machida et al., 2017), including multiple sclerosis (Zenaro et al., 2017) or AD (Winkler et al., 2014). Recent research also confirmed the association of the loss of pericytes and accumulation of fibrillar amyloid-β (Nikolakopoulou et al., 2017; Miners et al., 2018). Astrocytes are another NVU cell type that play a crucial role in the transport of amyloid-β across the BBB (Cai et al., 2017). It seems that their dysfunction can lead to the increase of RAGE activity and decrease of the LRP-1 function (Sagare et al., 2007; Askarova et al., 2011). Endothelial cells of the BBB are strongly connected by tight junctions. Yamazaki et al. (2019) have shown that the loss of cortical tight junction proteins is a common event in AD, and is correlated with synaptic degeneration. To sum up, BBB dysfunction can induce tau hyperphosphorylation, and vice versa, tau pathology can trigger the BBB damage (Ramos-Cejudo et al., 2018). Neuroinflammation and oxidative stress also contribute to the BBB damage and formation of NFTs (Ojala and Sutinen, 2017; Kumfu et al., 2018).

Cerebrovascular inflammatory changes are associated with AD pathology. iAnalysis of AD patients showed that cerebrovascular endothelium expressed increased levels of ICAM-1 and monocyte chemoattractant protein (MCP-1; Frohman et al., 1991; Grammas and Ovase, 2001). Compared to controls, AD microvessels produced significantly higher amounts of a number of inflammatory molecules such as TNF-α, transforming growth factor-β (TGF-β), nitric oxide, thrombin, cytokines such as IL-1β, IL-6, IL-8, and matrix metalloproteinases (MMPs; Grammas and Ovase, 2001). TGF-β1 as pro-inflammatory cytokine that negatively modulate vasculogenesis, angiogenesis, and vessel wall integrity. In AD patients, TGF-β1 has been associated with extracellular senile plaques and intracellular NFTs (van der Wal et al., 1993). Higher levels of TGF-β1 were found in serum and cerebrospinal fluid of demented AD patients compared to age-matched controls (Chao et al., 1994). The overexpression of TGF-β1 induced an accumulation of basement membrane proteins and led to cerebrovascular amyloidosis and vascular degeneration in Tg mice, confirming its important role in BBB changes (Wyss-Coray et al., 2000).

### Frontotemporal Dementia and Parkinsonism Linked to Chromosome 17

FTDP-17 is the second most common form of dementia with 20% of all dementia cases. FTDP-17 is clinically characterized by progressive behavioral, cognitive, and motor changes, including poor impulse control, inappropriate social conduct, apathy, worse cognitive control, and limited mental flexibility. These changes often precede the extrapyramidal and corticospinal motor signs and symptoms (Sitek et al., 2014).

Numerous cases of frontotemporal dementia show dynamic neuropathological changes caused by the abnormal deposition of protein tau (Schweitzer et al., 2006). FTDP-17 is a subtype of frontotemporal dementia, and it represents a group of neurodegenerative tauopathies caused by mutations in tau and progranulin genes (Hutton et al., 1998; Poorkaj et al., 1998; Spillantini et al., 1998; Sitek et al., 2014). Different mutations in the tau gene can affect tau mRNA splicing, altering the ratio of 3R and 4R tau (Spillantini and Goedert, 2013).

There are some evidence about microvascular changes in frontotemporal dementia. De Reuck et al. (2012, 2013) performed an examination of several post-mortem brains of patients with frontotemporal dementia and found microbleeds predominantly within the areas affected by neuronal pathology, suggesting the disruption of the BBB.

### PSP and Vascular PSP

PSP is a 4R tauopathy characterized by progressive deterioration of brain cells, mostly in the region of the brainstem. In addition, glial “tufted astrocytes” and neuronal tangles in gray matter and oligodendrocytic “coiled bodies” in the white matter of the neocortex are present (Irwin, 2016). The most common subtypes of PSP are Richardson’s syndrome (RS) and progressive supranuclear palsy-parkinsonism (PSP-P; Srulijes et al., 2011). Clinical features of RS include early gait instability, falls, supranuclear gaze palsy, axial rigidity, dysarthria, dysphagia, and progressive dementia. PSP-P is clinically characterized by tremor, rigid bradykinesia, levodopa responsive, late cognitive decline, and longer life expectancy. Among less common PSP syndromes belong PSP-pure akinesia with gait freezing, PSP-corticobasal syndrome, PSP-behavioral variant of frontotemporal dementia, PSP-primary lateral sclerosis, or PSP-cerebellar variant (McFarland, 2016).

The study characterizing the P*-*glycoprotein (P-gp) function at the BBB using [^11^C]-verapamil PET in PSP patients revealed increased [^11^C]-verapamil uptake in basal ganglia and frontal regions, suggesting decreased function of the transporter in these areas. Even if these results were not significant, they showed differences between the patients in various stages of the disease. These results are consistent with regionally decreased P-gp function with the progression of the disease (Bartels et al., 2008).

Vascular PSP is a multi-infarct disorder presenting as PSP (Josephs et al., 2002). This rare akinetic-rigid syndrome is characterized by asymmetric lower-body involvement, predominant corticospinal and pseudobulbar signs, urinary incontinence, cognitive impairment, increased frequency of stroke risk factors, and neuroimaging evidence of vascular changes in subcortical regions, especially the bilateral frontal lesions (Lanza et al., 2014). Vascular PSP differs from idiopathic PSP by a higher degree of asymmetry, lower body involvement, and evidence of corticospinal and pseudobulbar signs (Winikates and Jankovic, 1994).

### CBD and Vascular Corticobasal Syndrome

CBD is a rare, progressive neurodegenerative 4R tauopathy associated with heterogeneous motor, sensory, behavioral, and cognitive symptoms. CBD pathology is characterized by circumscribed cortical atrophy with spongiosis and ballooned neurons. Tau pathology is extensively present in neurons and glial cells of the gray and white matter of the cortex, basal ganglia, diencephalon, and rostral brainstem. Abnormal tau accumulation within astrocytes forms pathognomonic astrocytic plaques. The classic clinical presentation, corticobasal syndrome, is characterized by asymmetric progressive rigidity and apraxia with limb dystonia and myoclonus (Kouri et al., 2011). The corticobasal syndrome also accompanies other diseases, including AD and PSP. Moreover, the pathology of CBD can be associated with Richardson syndrome, behavioral variant of frontotemporal dementia, primary progressive aphasia and posterior cortical syndrome (Kouri et al., 2011).

Recently, a few cases of vascular corticobasal syndrome (CBS) have been reported. Koga et al. (2019) have investigated 217 patients with an antemortem diagnosis of CBS and among them, they identified three patients with vascular CBS. Multiple infarcts in the frontal lobe and motor cortex, periventricular white matter, thalamus, and basal ganglia were observed in two patients. One patient had no cortical infarct but had multiple white matter infarcts and corticospinal tract degeneration. This autopsy study showed that, while rare, cerebrovascular pathology can underlie clinical features suggestive of CBS (Koga et al., 2019).

### Chronic Traumatic Encephalopathy

In the past, CTE was referred to as dementia pugilistica. The recent studies have shown neuropathological evidence of CTE in retired American football players, professional wrestlers, professional hockey or soccer players, as well as in nonathletes. CTE may have different causes, such as falls, motor vehicle accidents, assaults, epileptic seizures, or military combat (Gavett et al., 2010). However, not all patients with repetitive brain trauma develop CTE, indicating that additional risk factors, including genetics, may play a role in the neuropathogenesis of this disease. It has also been suggested that the APOe3 allele may increase susceptibility for CTE (Gandy and Dekosky, 2012; Stern et al., 2013). In CTE, both 3R- and 4R-tau isoforms are present in neurofibrillary pathology (Woerman et al., 2016). CTE is clinically manifested by impairments in cognition, behavior, and mood, and in some cases, chronic headache and motor and cerebellar dysfunction are occasionally accompanied by dizziness and headaches (McKee et al., 2009; Stern et al., 2013). Previous microvasculature studies of several dementia pugilistica cases revealed decreased microvascular density and tortuosity with a strong correlation between the laminar distribution of NFTs and pathological microvasculature. It has been suggested that repetitive head trauma may cause vascular damage with the subsequent NFTs and neuropil neurites formation in perivascular space (McKee et al., 2009). A recent study proved the BBB disruption mainly in regions of intense perivascular tau deposition. The accumulation of tau protein was associated with loss of the tight junction protein claudin-5 and enhanced extravasation of endogenous blood components such as fibrinogen and IgG (Farrell et al., 2019).

### Parkinsonism Dementia Complex of Guam (Lytico-Bodig Disease)

PDCG belongs to rare tauopathies. It is a disorder unique to the Chamorro people of Guam and the Mariana Islands. Strong familial clustering suggests the genetic origin of the disease (Hirano et al., 1961a,b; Schwab et al., 1999). Parkinsonism, dementia, or a combination of both are found as initial symptoms; however, most PDC patients show a gait disturbance with additional extrapyramidal symptoms.

Patients with PDC shows recent memory deficits, disorientation in time and place, behavioral changes, and progressive deterioration of all intellectual skills. Over time, they reach a bedridden state (Chen, 1985). Other clinical features include olfactory dysfunction and, in some individuals, oculomotor signs (Kovacs, 2015).

Neurofibrillary pathology similar to that observed in AD, but without the presence of amyloid plaques is found in most PDC cases (Lee et al., 2001).

Previous studies described brain microvascular changes in PDC. They found a decrease in vascular density, atrophic, and fragmented microvessels with a reduced number of microvascular branches mainly in the areas affected by NFTs pathology (Buee et al., 1994, 1997). Recently, we showed that neurofibrillary pathology is closely associated with cerebrovascular inflammatory changes in Guam PDC patients. The areas with significant accumulation of the tau in the NFTs correlated with upregulation of adhesion molecules, disruption of tight junctions, morphological alterations in brain microvessels such as thickening of the vessel walls, and narrowing of the vessel lumens and an increase in collagen-type IV content per vessel (Majerova et al., 2018).

### Pick’s Disease

PiD is a 3R-tau predominant tauopathy characterized by the presence of “Pick bodies” comprising the aggregates of hyperphosphorylated tau and glial inclusions through the limbic and neocortical regions (Irwin, 2016). Moreover, ramified astrocytes are present (Komori, 1999). Severe neuronal and glial loss in PiD leads to frontotemporal lobe atrophy, and it is clinically manifested by the loss of verbal skills, personality changes, and progressive dementia (Hardin and Schooley, 2002; Rohn et al., 2013). Vascular changes in PiD, including thinning of microvessels, increased tortuosity, twisted vessels, and fragmentation of microvasculature. The structural changes of capillaries are comparatively severe as in AD (Buee et al., 1997). Massive disorganization of the laminar distribution of microvessels is shown mainly in atrophy-affected areas (Buee et al., 1994).

### Argyrophilic Grain Disease

AGD is a highly frequent but still under-recognized neurodegenerative condition. AGD is a sporadic 4R tauopathy. In the past, AGD was reported as adult-onset dementia, but extended studies have revealed clinical features, such as changes of the personality, emotional imbalance, or memory problems. Pathologically, AGD is characterized by the presence of spindle-shaped or comma-shaped argyrophilic grains in the neuropil of the entorhinal cortex, hippocampus, and amygdala (Togo et al., 2002).

### Globular Glial Tauopathies

GGTs represent a group of 4R tauopathies that are characterized neuropathologically by widespread globular glial inclusions. These tauopathies are very rare and they have a range of clinicopathological presentations. We can divide them into three main types—type I cases are typically presented with frontotemporal dementia correlating with the frontotemporal distribution of pathology, type II cases are predominately characterized by motor cortex and corticospinal tract degeneration, and type III cases can present with a combination of the frontotemporal, motor cortex, and corticospinal tract involvement. Extrapyramidal features can be present in types II and III, and in all types of globular glial tauopathies, significant degeneration of the white matter can be observed (Ahmed et al., 2013). It is known that astrocytes play a key role in maintaining the BBB *via* astrocytic endfeet, which are directly opposed to vascular endothelial cells (Ransom et al., 2003). Experiments on mice confirmed the mild BBB disruption after the targeted expression of human wild-type tau in murine astrocyte (Zlokovic, 2008; Bartels et al., 2009). In addition, astrocytes also modulate synaptic function by the secretion and uptake of neurotransmitters such as glutamate, the brain’s major excitatory neurotransmitter (Haydon, 2001).

### Aging-Related Tau Astrogliopathy

Aging-related tau astrogliopathy is characterized by the presence of two types of tau-bearing astrocytes. The first type, thorn-shaped astrocytes, is located in the subependymal and subpial regions, perivascular spaces, and in clusters in the frontal and temporal cortices, basal forebrain, and brainstem. They were first described in association with AD and AGD. The second type is represented by granular/fuzzy astrocytes, which are mainly located in the gray matter, and they were firstly identified in a particular subgroup of patients with dementia. Both types can also be present in combination with other tauopathies (Ferrer et al., 2018). The etiology of this disease remains unclear; however, functional changes of the BBB together with metabolic encephalopathy, neurodegenerative pathologies, aging-related hypoperfusion, AD, vascular dementia, and even repeated minor trauma with possible genetic risk factors may play a role (Kovacs et al., 2016).

## Clinical Challenges

As discussed in previous sections, the neurofibrillary pathology is connected with changes at the neurovascular unit. Moreover, the distribution and load of neurofibrillary pathology is correlated with clinical phenotype and severity of cognitive impairment (Nelson et al., 2012; Murray et al., 2015).

Clinical studies suggest that the NVU changes may represent an early biomarker of human cognitive dysfunction (Nation et al., 2019). The [^18^F]Fluoro-2-deoxy-d-glucose (FDG-PET)/CT studies suggest that in about 16% of analyzed AD cases, BBB dysfunction may be present, and this pattern is related to a worse metabolic pattern (Chiaravalloti et al., 2016). Montagne et al. (2020) performed the analysis of the BBB permeability in 245 participants using the dynamic contrast-enhanced magnetic resonance imaging. They showed increased BBB permeability in cognitively normal APOε4 carriers, compared to cognitively normal APOε3 homozygotes, both with clinical dementia rating scores of 0. This increase was independent of amyloid-β and tau levels, indicating APOε4 to be a potential therapeutic and diagnostic target in APOε4 carriers (Montagne et al., 2020).

However, major questions remain, such as how tau-induced NVU changes are presented at the clinical level and whether early biomarkers of these processes could help in more focused therapeutic intervention in the future.

## Author Contributions

AM, PM, and AK wrote the manuscript. All authors read and approved the final draft of the manuscript. All authors contributed to the article and approved the submitted version.

## Conflict of Interest

The authors declare that the research was conducted in the absence of any commercial or financial relationships that could be construed as a potential conflict of interest.
